# Sunday Pig Dinners

**Published:** 1859-11

**Authors:** 


### SUNDAY PIG DINNERS.

Poughkeepsie, August 6th, 1856.

Dear Sir:

Since I last saw you, I have been in almost uninterrupted health; my strength was so great, that I could labor with any man at any kind of work all day, and feel best at night, and during the hot days I did most and felt best. This, I suppose, caused a carelessness in diet, though I hardly yet am conscious of it, for I seldom over-eat, though I did Sunday before last—that hot Sunday—eat such a dinner as I would have done on Monday, and then yielded to the influence of the day, there being no service in church in the afternoon, and laid down on the floor and slept till tea-time. Monday morning, I awoke at 4 o'clock, with a profuse painless diarrhœa, and by 3 o'clock, P.M., I was passing a white, watery fluid, and passed more water than I had drank in a fortnight.

We are in a state of betweenity entirely, as to whether we are under most obligation to ask pardon of a pig or our correspondent for the heading we have given to his letter. We are rather inclined to vote for the pig. Pigs don't eat "*Sunday dinners.*" They treat every day alike, and eat every day, at least, when they can get it, until they don't want to eat any more. Man is the only animal in nature who eats more than he wants, as far as our knowledge of natural history informs us.

We have much less muscular and mental exercise on Sundays than on week days, hence, much less wear and tear and waste, therefore a proportionably less need for supply for repairs. Multitudes make the dinner for Sunday the most sumptuous of the week, with artificial tempters of the appetite to boot. It is no wonder, then, that on Sunday afternoon, we are a nation of gorged anacondas, too sleepy to see, too stupid to think, and too lazy to move.

The best Sunday dinner—best for the body, best for the soul—is half a glass of water, a piece of bread and butter, and a slice of cold meat. Thus doing, while our servants will bless us, our ministers will preach to listening congregations and not to sleeping hulks.

Meanwhile, if any one desires at any time to get up an extemporaneous *amateur* cholera-morbus performance, let him take a nap on the floor soon after a hearty dinner. The lowest part of a room being the coldest, a person sleeping gets chilled on the outside, checking all exhalations and driving the drains inwards, to find vent in the manner just named. To clergymen who officiate in the morning and afternoon, the advice is given to eat nothing at all beyond an apple or two or orange, or piece of bread and butter, with a cup of warm drink between the services, they will thus be able to preach easier to themselves and more profitably to their hearers, because it requires less physical effort, and the mind is more active.

We personally know two western clergymen, equally eminent, and deservedly occupying positions of high responsibility—one eats nothing between the services, the other dines on strong tea—the former we have never known to be ill, the latter has a dangerous attack of sickness every year or two, and is very often complaining between times.

